# Physiological responses of Goji berry (*Lycium barbarum* L.) to saline-alkaline soil from Qinghai region, China

**DOI:** 10.1038/s41598-019-48514-5

**Published:** 2019-08-19

**Authors:** Zhenzhong Zhang, Kangning He, Tan Zhang, Da Tang, Runjie Li, Shaofeng Jia

**Affiliations:** 1 School of Soil and Water Conservation, Beijing Forestry University, Beijing, China; 2 Key Laboratory of State Forestry Administration on Soil and Water Conservation, Beijing Forestry University, Beijing, China; 3 Beijing Engineering Research Center of Soil and Water Conservation, Beijing Forestry University, Beijing, China; 4 Engineering Research Center of Forestry Ecological Engineering of Ministry of Education, Beijing Forestry University, Beijing, China; 5 Institute of Geographical Sciences and Natural Resources Research, Chinese Academy of Sciences, Beijing, China; 6 Key Laboratory of Water Cycle and Related Land Surface Processes, Chinese Academy of Sciences, Beijing, China; 7 Present Address: Power China Guiyang Engineering Corporation limited, Guiyang, Guizhou Province China; 8 Institute of Water Resources and Hydropower of Qinghai Province, Xining, Qinghai Province China

**Keywords:** Homeostasis, Homeostasis, Forestry, Forestry

## Abstract

Recently, Goji berry (*Lycium barbarum* L.) has been extensively cultivated to improve the fragile ecological environment and increase the income of residents in Qinghai Province, northwestern China. However, few studies have focused on the physiological responses of Goji berry under salt stress and alkali stress. Gas exchange, photosynthetic pigments, and chlorophyll fluorescence were evaluated in response to neutral (NaCl) and alkali (NaHCO_3_) salt stresses. Nine irrigation treatments were applied over 30 days and included 0(Control group), 50, 100, 200, and 300 mM NaCl and NaHCO_3_. The results showed that salt and alkali stress reduced all the indicators and that alkali stress was more harmful to Goji berry than salt stress under the same solution concentrations. The salt tolerance and alkali resistance thresholds were identified when the index value exceeded the 50% standard of the control group, and threshold values of 246.3 ± 2.9 mM and 108.4.7 ± 2.1 mM, respectively, were determined by regression analysis. These results were used to identify the optimal water content for Goji berry. The minimum soil water content to cultivate Goji berry should be 16.22% and 23.37% under mild and moderate salt stress soils, respectively, and 29.10% and 42.68% under mild and moderate alkali stress soil, respectively.

## Introduction

Salt-alkalization is becoming an increasing environmental and socioeconomic problem because it leads to the loss of agricultural land at a rate of 0.25 to 0.5 Mha annually around the world^1^. Alkaline soils are primarily caused by the accumulation of NaHCO_3_, whereas saline soils are mainly due to NaCl accumulation^2,3^. In China, especially northwestern China, overgrazing, evaporation, overirrigation, and deforestation are crucial factors that result in severe secondary salinization, which can significantly reduce crop productivity^4–6^. Conventional engineering salt washing has many deficiencies, such as high costs, water waste, and side effects. Therefore, phytoremediation with native plants which has a high salt and alkali tolerance is a practical solution from an economic and environmental perspective^7,8^. Generally, salt stress in the form of NaCl is associated with imbalances in ion levels and homeostasis^7,9^, leading to oxidative stress and ion overload^10–12^. In contrast, alkali stress is mainly caused by excess NaHCO_3_ in the soil solution that results in a high pH. Recent studies have shown that salt stress can cause damage to physiological processes, including the photosynthetic apparatus, chlorophyll fluorescence, osmotic adjusting materials and antioxidant defence mechanisms^13^. In recent years, studies have primarily focused on plant responses to salt stress, while little attention has been paid to the effect of alkalinity, especially the severe effects of alkalinity compared to salinity^14^. Hence, understanding the physiological response of plants to alkalinity is of great significance for selecting adaptive crop plants with increased resistance to alkalinity.

Photosynthesis is of paramount importance and represents a physiological process that can be inhibited by salt or alkali stress^15,16^. Researchers have indicated that stomatal closure is the main factor that decreases the assimilation ability of CO_2_ under salt stress and results in a decline in photosynthetic capacity^17^. Salt stress also has an influence on photosynthetic components and chloroplast ultrastructure^18,19^, which are believed to be nonstomatal factors that affect the decline in photosynthetic capacity^20,21^. Chlorophyll (Chl) fluorescence represents a sensitive and noninvasive probe and has been used to study the function and performance of the photosynthetic machinery of various plants, and several recent works have shown that this tool can be useful for identifying stress factors that affect or/and limit plant growth^22^. However, the effects of alkali stress on photosynthesis, especially on Chl fluorescence, are poorly understood. Malondialdehyde (MDA), superoxide dismutase (SOD), peroxidase (POD) and catalase (CAT) play paramount roles in osmotic adjustment and may also be of great importance in eliminating reactive oxygen species (ROS) when plants are under salt stress^23^. However, the relationship between antioxidant enzymes and alkali stress has not been clarified.

Qinghai Province in China is located in an extremely cold region—the transition zone between the Tibetan Plateau and the Loess Plateau. Qinghai suffers from serious soil salt-alkalization and has a salt-alkali area of 15,720 km^2^, which accounts for 2.3% of the total area of China according to the work of Cai^24^. The most severe salinization in Qinhai Province is centralized in Qaidam basin. The main ion types of this area are Na^+^, Cl^−^, and HCO_3_^−^, and the ion density of each ion is approximately 2.71, 7.67 and 0.28 g kg^−1^, respectively. Saline or alkali soils seriously affect the vegetation cover and ecological environment, as well as affecting the land use status, thus influencing the economic development of the entire Qinghai Province. Based on the geo-ecological uniformity, an effective method for ameliorating the fragile ecological environment in Qinghai Province is to improve soil salt-alkalization by cultivating abundant native plants that are highly capable of acclimating to and improving the adverse soil conditions^25^.

Goji berry (*Lycium barbarum* L.), which is also named Wolfberry, is a perennial plant in arid areas that is used in traditional herbal medicine. The Goji berry is usually consumed for dietetic and medicinal purposes, as its leaves are rich in flavonoids, polysaccharides, and amino acids^26^. Recently, interest in Goji berry has greatly increased, and the role of Goji berry in local economies has become increasingly important in recent years. Goji berry has also been proposed as a potential pioneer plant to reclaim salinized and alkalinized soils because Goji berry growth and photosynthesis have been shown to be negatively affected by high levels of salt stress^27^. However, previous studies on the effects of salt stress on Goji berry have primarily focused on physiological changes under salt stress, and the response to alkali stress has not been sufficiently studied.

Considering the abovementioned issues, the specific objectives of this study were to investigate the variations in leaf gas exchange, Chl content, Chl fluorescence and antioxidant enzymes in Goji berry under salt and alkali stress. In addition, we performed a regression analysis to determine the salt tolerance and alkali tolerance threshold of Goji berry, which was used to establish the correlation between the saline-alkali content and soil moisture content. Our results regarding the parameters mentioned above will advance our understanding of the mechanisms of salt and alkali tolerance of Goji berry and provide a scientific basis for the field production and water management of Goji berry under different saline-alkali conditions in Qinghai Province.

## Results

### Effects of salt stress and alkali stress on gas exchange

**Figure 1:**
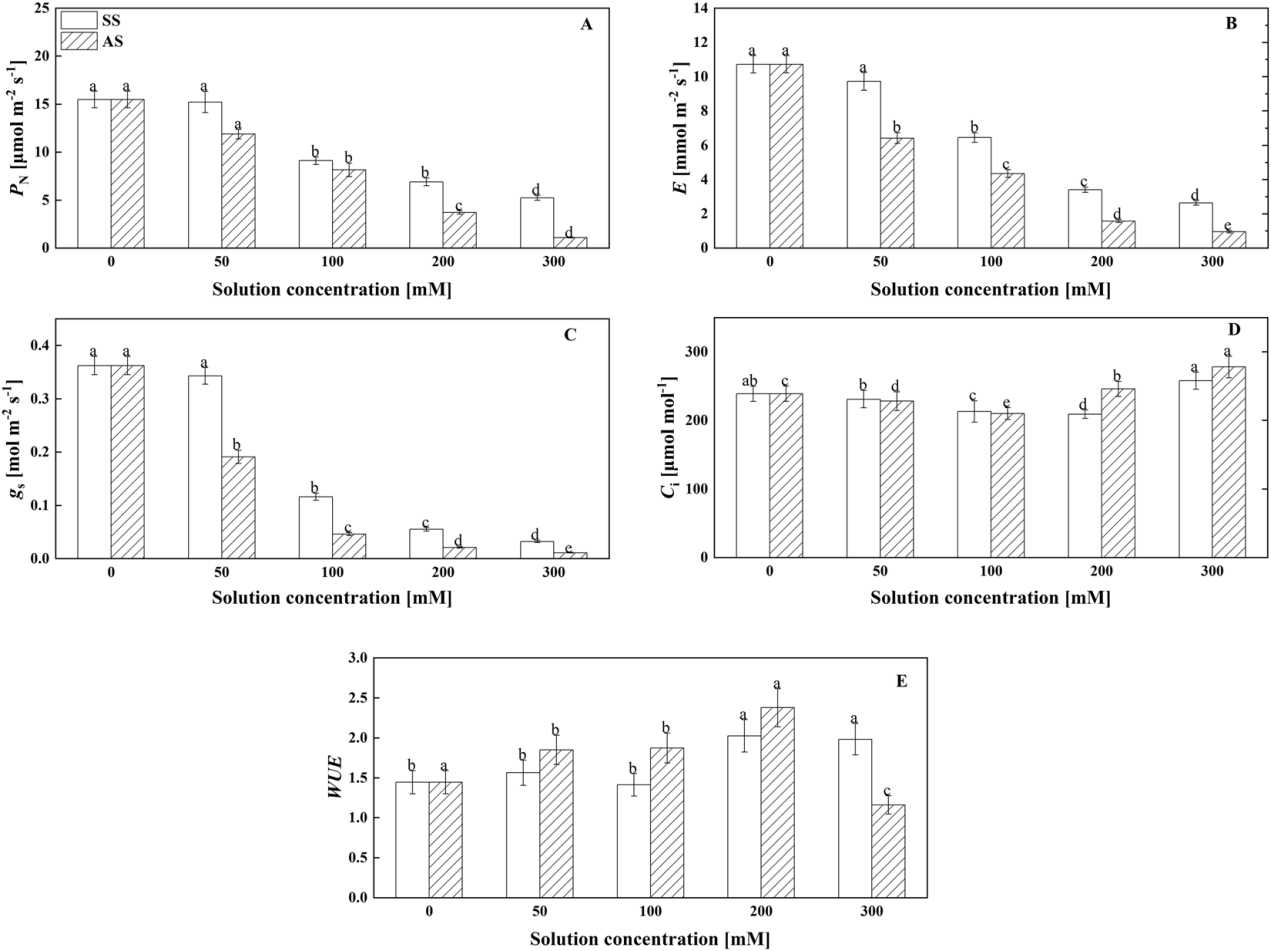
Effects of NaCl and NaHCO_3_ concentrations on the (**A**) net photosynthetic rate (P_N_), (**B**) transpiration rate (E), (**C**) stomatal conductance (g_s_), (**D**) intercellular CO_2_ concentration (C_i_) and (**E**) water use efficiency (WUE) in the leaves of Goji berry. Values are represented as the mean ± SE (n = 5). Different letters indicate a significant difference at P < 0.05 by the least significant difference (LSD) test.

As shown in Fig. 1, 50 mM NaCl did not have a significant effect on the net photosynthetic rate (P_N_), transpiration rate (E), stomatal conductance (g_s_) or intercellular CO_2_ concentration (C_i_), whereas 50 mM NaHCO_3_ had a significant effect on the values of these parameters (P < 0.05, Fig. 1). With increasing concentrations of salt or alkali (greater than 50 mM), the P_N_, E and g_s_ values apparently decreased, and the extent of the reduction under alkali stress was greater than that under salt stress (P < 0.05, Fig. 1). However, the C_i_ value exhibited a constant decreasing trend at salinity levels ranging from 0~200 mM, and the value increased at a salinity level of 300 mM (P < 0.05, Fig. 1D). The trend exhibited by C_i_ under alkali stress was similar to that exhibited under salt stress, although the value started to increase at 200 mM (P < 0.05, Fig. 1D). The water use efficiency (WUE) of Goji berry under both salt stress and alkali stress showed an increasing trend with an increasing concentration of salt or alkali (P < 0.05, Fig. 1E). Interestingly, the WUE of Goji berry under 300 mM alkali stress was extremely lower than other alkali treatments.

### Effects of salt stress and alkali stress on the chlorophyll pigment content

**Figure 2:**
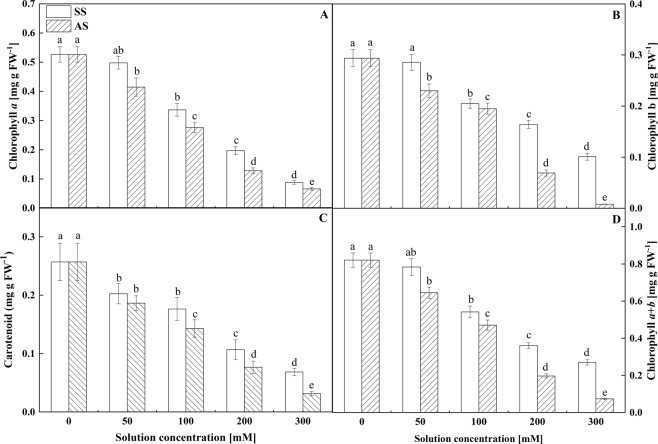
Effects of salt stress (SS) and alkali stress (AS) on the contents of (**A**) chlorophyll a, (**B**) chlorophyll b, (**C**) carotenoids and (**D**) chlorophyll (a + b). Means values indicated by different letters in the same curve are significantly different at P < 0.05 according to the least significant difference (LSD) test.

Differences were not observed in the Chl pigment content of Goji berry between the low-concentration (50 mM) salt treatment and the control. However, salt stress markedly decreased the Chl a, Chl b, carotenoids (Cars) and Chl (a + b) values in the leaves at salinity levels higher than 100 mM (P < 0.05, Fig. 2). Plants treated with NaHCO_3_ exhibited a constant decrease in Chl pigment content, including the Chl a, b, Cars and (a + b) values (P < 0.05, Fig. 2), and the rate of decrease was greater than that observed under salt stress treatment at the corresponding concentrations. Maximum reductions in Chl a, b, Cars and (a + b) values (67.74, 65.65, 73.35 and 66.70%, respectively) were observed in the 300 mM salt stress treatment, whereas these values were 89.18, 94.22, 87.72 and 90.99% in the 300 mM alkali stress treatment. In general, the Chl a and b values were more sensitive (or less resistant) to alkali stress than salt stress at the same concentrations, which caused a distinct decrease in the Chl (a + b) value (P < 0.05, Fig. 2).

### Effects of salt stress and alkali stress on the chlorophyll fluorescence

**Figure 3:**
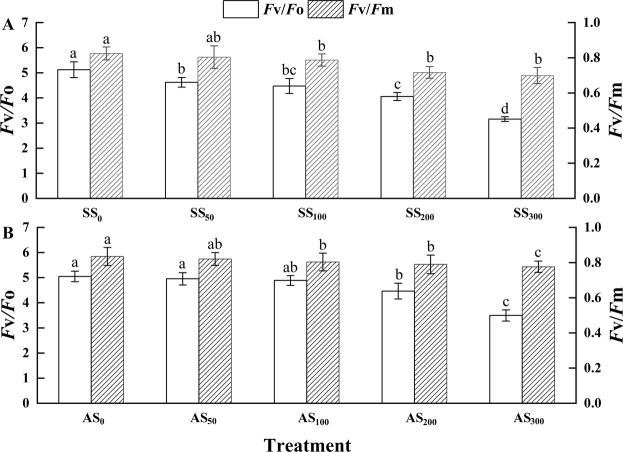
Effects of (**A**) NaCl and (**B**) NaHCO_3_ on the F_v_/F_m_ and F_v_/F_o_ values of Goji berry after 20 days of treatment with different concentrations. Values are represented as the mean ± SE (n = 5). Abbreviations: Fv/Fm: maximum quantum yield of electron transfer of PSII, Fv/Fo: activity of the water-splitting complex.

As shown in Fig. 3, salt stress decreased the maximum quantum yield of PSII (F_v_/F_m_) value by 3.16, 11.58 and 30.76% compared to the value of the control group in Goji berry subjected to 100, 200 and 300 mM, respectively (P < 0.05). On the other hand, salt stress decreased the F_v_/F_o_ values by 5.82, 15.96, 24.91 and 34.48% compared to the value of the control group when the seedlings were subjected to 50, 100, 200 and 300 mM salinity, respectively (P < 0.05, Fig. 3A). Furthermore, a magnitude of F_v_/F_m_ greater than 0.78 was observed in only the group treated with the 50 mM salt solution. Under alkali stress, the F_v_/F_m_ value was found to decrease by 44.35 and 55.15%, (P < 0.05, Fig. 3B) when treated with 200 and 300 mM, while the F_v_/F_o_ value was found to decrease by 20.79 and 38.35% in Goji berry treated with 200 and 300 mM alkali solution, respectively (P < 0.05, Fig. 3B).

**Figure 4:**
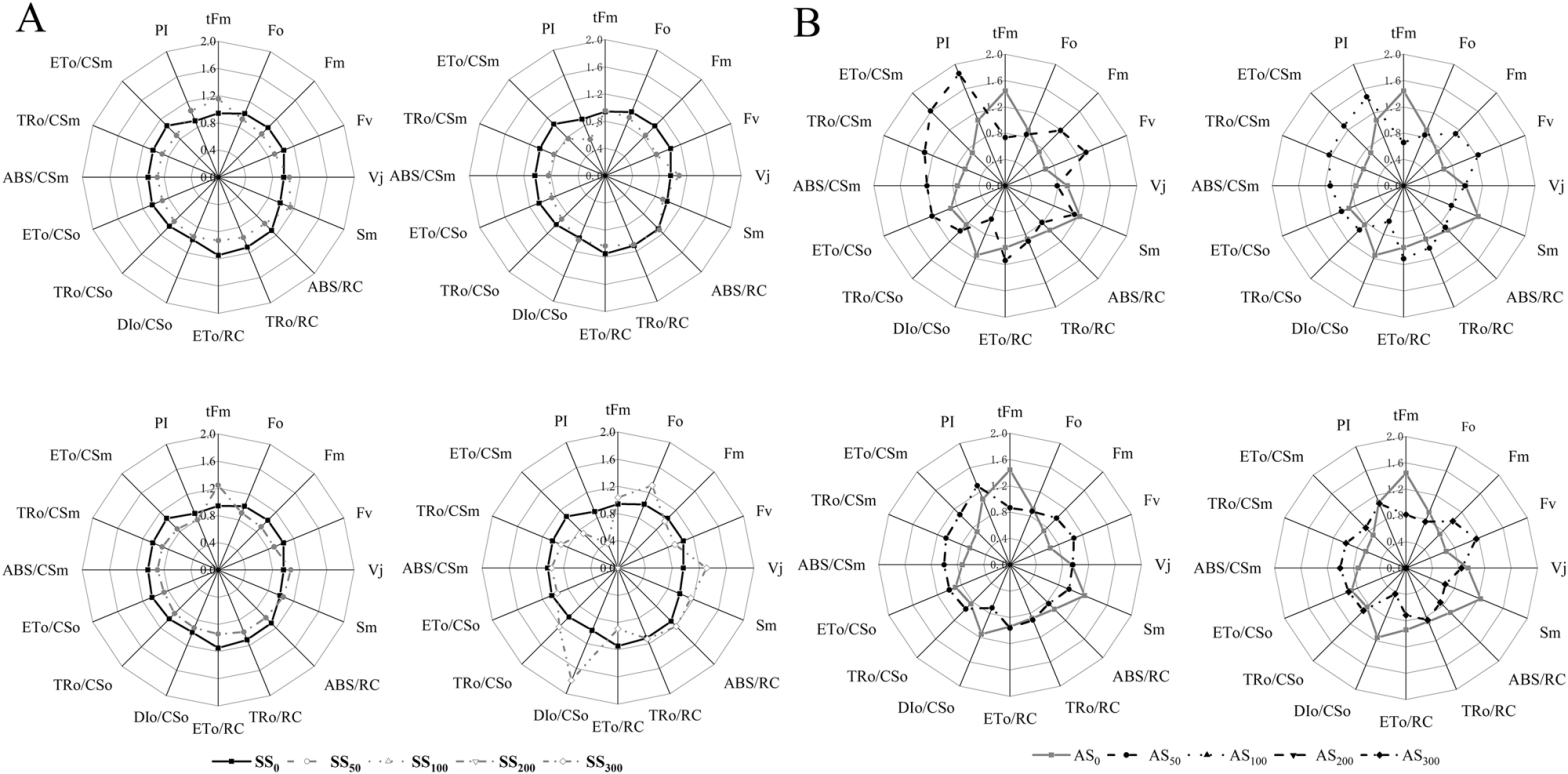
Effects of (**A**) salt stress and (**B**) alkali stress on the parameters associated with the chlorophyll fluorescence of Goji berry after 20 days of treatment with different salt solutions. Values are represented as the mean ± SE (n = 5).

In terms of the parameters associated with Chl fluorescence, salt stress increased the nonvariable Chl fluorescemce level (Fo), relative variable fluorescence at the J step (Vj), reductions of QA (Sm), absorption energy flux (ABS/RC), energy dissipation in the unit reaction center (DIo/CSo) and energy capture in the unit reaction center (TRo/CSo) values by 29.7, 35.53, 17.72, 12.28, 80.09 and 19.89%, respectively (P < 0.05, Fig. 4A). However, salt stress decreased the variable fluorescence (Fv), ETo/RC, TRo/CSm, ETo/CSm and performance index of intersystem electron acceptors (PI) values by 12.77, 22.20, 12.78, 32.74 and 56.20%, respectively (P < 0.05, Fig. 4A). In addition, salt stress had no significant effect on the time to reach maximum fluorescence intensity (tFm), maximum fluorescence induction (Fm), TRo/RC, ETo/CSo and ABS/CSm values (P > 0.05, Fig. 4A).

When Goji berry was subjected to 300 mM alkali solution, the alkali stress increased the Fm, Fv, Vj, ABS/CSm, TRo/CSm, and ETo/CSm values by 38.88, 74.94, 50.77, 37.75, 49.16 and 22.15%, respectively (P < 0.05, Fig. 4B). However, alkali stress decreased the tFm, Fo, Sm, ABS/RC, ETo/RC, and DIo/CSo values by 43.70, 16.93, 47.19, 22.60, 24.02 and 62.69%, respectively (P < 0.05, Fig. 4B). In addition, no significant difference was observed in the TRo/RC, TRo/CSo, ETo/CSo and PI values (P > 0.05, Fig. 4B).

### Effects of salt stress and alkali stress on antioxidant enzyme activities

**Figure 5:**
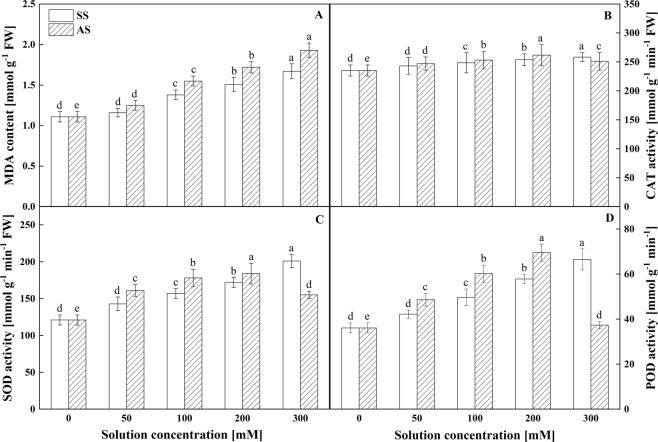
Effects of NaCl and NaHCO_3_ concentrations on (**A**) malondialdehyde (MDA) levels, (**B**) catalase (CAT) activity, (**C**) superoxide dismutase (SOD) activity, and (**D**) peroxidase (POD) activity in the leaves of Goji berry. Values are represented as the mean ± SE (n = 5). Different letters indicate a significant difference at P < 0.05 by the least significant difference (LSD) test.

Significant differences were not observed in the MDA content at a 50 mM salinity level (P < 0.05, Fig. 5A); however, the MDA content increased significantly with increasing salinity. The subsequent assays suggested that the MDA content in the leaves was higher under alkali stress than under salt stress. In addition, seedlings under alkali stress exhibited the same increasing tendency as those under salt stress (P < 0.05, Fig. 5A). Under both types of stress, the MDA levels in the seedlings plateaued in the 300 mM treatment (Fig. 5A).

The results of the CAT activity assay showed that CAT increased in a linear manner under salt stress at salinity levels ranging from 50 to 100 mM (P < 0.05, Fig. 5B). Under alkali stress, the CAT activity increased until the alkali concentration reached 200 mM, and then, the CAT activity decreased (P < 0.05, Fig. 5B). Compared with the control, the CAT activity under alkali stress was higher than that under salt stress (P < 0.05, Fig. 5B).

The protective enzyme activity determinations revealed that the SOD and POD activities in the leaves were similar under both types of stress. In general, the variation trend of POD activity was similar to that of SOD activity in response to salt or alkali stress. Compared with the control, salt stress significantly increased the SOD and POD activities, whereas alkali stress increased the SOD and POD activities at alkali concentrations ranging from 50 to 200 mM and then decreased these activities at the concentration of 300 mM (P < 0.05, Fig. 5C,D).

### Salt tolerance and alkali tolerance evaluation of Goji berry

Table 1 shows the correlations between the concentration, photosynthetic traits, and antioxidant attributes. P_N_ is significantly and positively correlated with g_S_, whereas P_N_ is significantly and negatively correlated with Ci. As Table 1 shows, P_N_ and SOD have the highest correlation coefficient with concentration. Therefore, P_N_ and SOD were selected to characterize the salt and alkali tolerance of Goji berry. P_N_ and SOD were separately fitted to the saline-alkali concentration, and the salt concentration of each index exceeding 50% of the control group was used as the salt tolerance and alkali threshold. The calculated thresholds of salt stress on P_N_ and SOD were 246.3 ± 2.9 and 254.3 ± 2.1 mM, respectively (P < 0.05, Table 2), and the lower value of 246.3 ± 2.1 mM was used as the salt tolerance threshold. On the other hand, the thresholds of alkali stress on P_N_ and SOD were 183.4 ± 3.4 and 108.4 ± 2.1 mM, respectively (P < 0.05, Table 2), and the lower value of 108.4 ± 2.1 mM was used as the alkali tolerance threshold.

**Table 1:** Coefficients of correlation (*r*^2^) among concentration, photosynthetic traits and antioxidant attributes of *Lycium barbarum* grown under various NaCl and NaHCO_3_ concentrations. Abbreviations: Chl: chlorophyll, P_N_: net photosynthetic rate, g_S_: stomatal conductance, E: transpiration rate, C_i_: intercellular CO_2_ concentration, MDA: malondialdehyde, SOD: superoxide dismutase, POD: peroxidase, CAT: catalase. **Significant correlation (P < 0.01, n = 20).

	Concentration	Chl	P_N_	g_S_	C_i_	E	MDA	SOD	POD	CAT
Concentration	1									
Chl	−0.95**	1								
P_N_	−0.93**	−0.93**	1							
g_S_	−0.84**	−0.81	−0.94**	1						
C_i_	0.86**	0.81	0.91**	0.49	1					
E	−0.89**	−0.85	−0.90	−0.94**	0.61	1				
MDA	0.96**	−0.90**	−0.95**	−0.95**	0.61	−0.96**	1			
SOD	0.78**	−0.90**	−0.95**	−0.93**	0.66	−0.90**	−0.78**	1		
POD	0.46	−0.83**	−0.84**	−0.89**	0.6	−0.97**	−0.41	−0.86**	1	
CAT	0.66	0.87	0.67	0.50	−0.78	0.59	−0.63	−0.93**	−0.90**	1

**Table 2:** Regression function of photosynthetic parameters, antioxidant system indices, and saline-alkali stress. Abbreviations: SOD: superoxide dismutase.

Treatment	Index	Regression function	R^2^	Thresholds
Salt stress	Net photosynthetic rate	y = −2e-05x^2^ −0.03x +15.695	R² = 0.91	246.3 ± 2.9
	SOD	y = −0.0002x^2^ +0.307x +124.52	R² = 0.89	254.3 ± 2.4
Alkali stress	Net photosynthetic rate	y = −8e-05x^2^ −0.1029x +50.726	R² = 0.94	183.4 ± 3.4
	SOD	y = −0.0022x^2^ +0.7524x +124.1	R² = 0.90	108.4 ± 2.1

### Linear regression of soli salt content and soil water content

**Figure 6:**
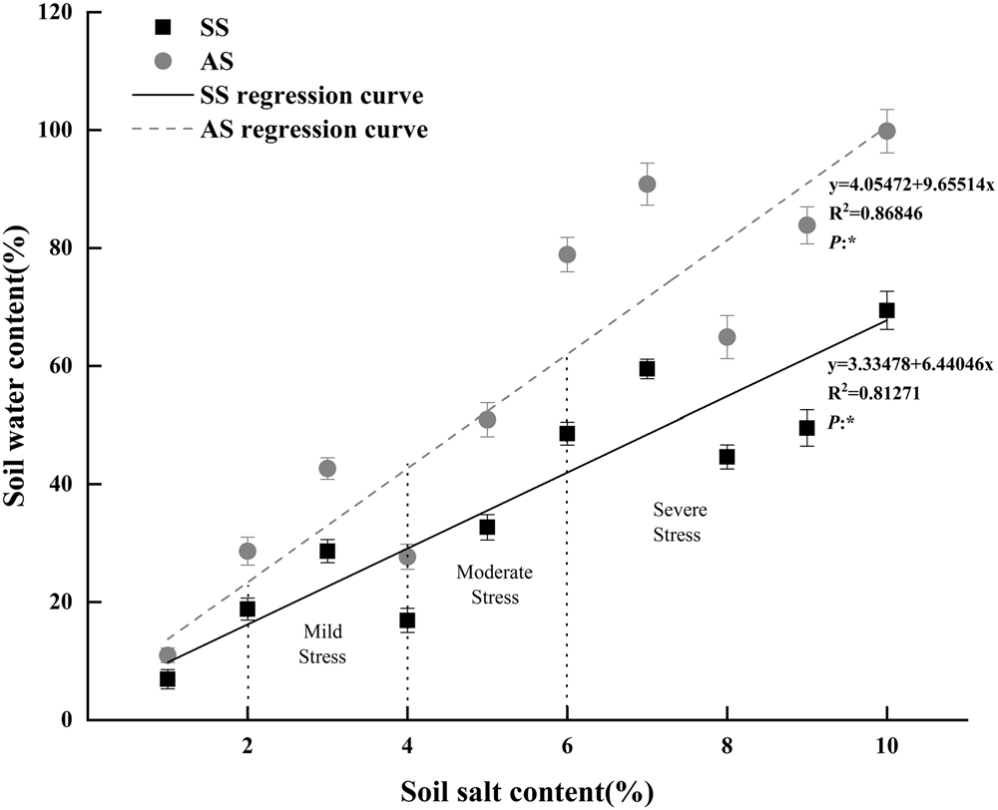
Linear regression curve of the soil salt content, and soil water content. *Means a significant difference at P < 0.05 (n = 5).

Based on the analysis of P_N_ and SOD activity of Goji berry under salt and alkali stress, the salt and alkali tolerance threshold range of Goji berry was determined to be 246.3 ± 2.9 mM and 108.4 ± 2.1 mM, respectively (P < 0.05, Table 2). The relationship between the salt content and water content of the local soil is shown in Fig. 6, and it is calculated based on the threshold of salinity (NaCl) and alkalinity (NaHCO_3_) of Goji berry. According to the Chinese criteria for the classification of soil salt-alkalization, a salt content of 2–4% indicates mild stress, 4–6% indicates moderate stress, and over 6% indicates severe stress^28^. As shown in Fig. 6, the minimum soil water content to cultivate Goji berry should be 16.22% and 23.37% in mild and moderate stress soils, respectively, and 29.10% and 42.68% in mild and moderate alkali stress soils, respectively.

## Discussion

The photosynthetic process is one of the major factors that controls plant growth under adverse conditions^29^. In the present experiment, P_N_, g_S_, and E decreased significantly in Goji berry under salt stress and alkali stress, which is consistent with previous reports of stress-induced limitations of photosynthetic capacity^26,30,31^. P_N_ was positively correlated with g_S_ under all stress levels (Table 1.), which could explain why the reduction in g_S_ caused a simultaneous decrease in both P_N_ and E. Such an inhibitory effect on photosynthesis has also been reported in another plant^32^. The reduction in P_N_ under salt and alkali stress is generally considered to be stomatal restriction caused by partial closure of stomata and decreased cell viability^33–35^. Moreover, the decrease in Ci and g_S_ were also the main reasons for the reduction of P_N_^36^. Yan *et al*.^37^ hypothesized that stomatal factors that inhibit photosynthetic activity are observed under conditions of intermediate salinity and alkalinity while nonstomatal factors function at high salinity and alkalinity. Moreover, the decrease in P_N_, g_S_, and E was even greater under alkali stress than under salt stress (Fig. 1A–C). Although salt stress and alkali stress significantly reduce the photosynthesis and transpiration rates of Goji berry, the water use efficiency in this species was enhanced, which is in accordance with the findings in *Kosteletzfkya virginica* L. Presl and *Quercus aliena* by Yue *et al*. and Farissi *et al*.^38,39^. It’s interesting that the water use efficiency of Goji berry under 300 mM alkali treatment was extremely lower than the other alkali treatment, which means that the alkali-tolerant threshold of Goji berry may be under 300 mM. This phenomenon showed that high pH induced by alkaline conditions might stimulate Goji berry roots to generate physical or chemical signals to affect stomatal opening and closing and gas exchange. Thus, the values of these photosynthetic parameters likely indicate the adaptability of Goji berry, and the values are far lower under alkali stress than under salt stress, which suggests that Goji berry seedlings are less adaptable to alkali stress than salt stress.

The Chl content is a crucial indicator of the status of chloroplasts. Chloroplast is the most important photosynthesizing organelles in plant, play an important role in the absorption and transformation of light energy and are extremely sensitive to adverse conditions. The concentration of Chl was decreased under both stresses. Under salt stress, the Chl concentration may have decreased because the capacity to compartmentalize adverse ion away from the chloroplasts was exceeded and the structures of the grana lamellae and thylakoids in the chloroplasts were damaged, which ultimately resulted in the reduction in the Chl concentration^40,41^. Both treatments added the same amount of Na^+^, although alkali stressed seedlings were affected to a greater degree, which may have been related to the precipitation of metal ions and phosphorus together with the disruption of ionic balance and pH homeostasis in plant tissues under alkali stress^42,43^.

We found that salt stress induced a decline in the F_v_/F_o_ and F_v_/F_m_ values by 34.48 and 30.76%, respectively, in Goji berry treated with 300 mM salt solution, whereas alkali stress induced a reduction in the F_v_/F_o_ and F_v_/F_m_ values by 38.35 and 55.15%, respectively, in Goji berry treated with 300 mM alkali solution. These results are in accordance with the report by Oukarroum *et al*.^44^ and are caused by the disruption of electron transport function in photosynthesis and an increase in the number of inactive RCs which results in the inhibition of the QA reduction process and can also lead to the same result under severe stress^45^. Furthermore, the values of F_v_/F_m_ greater than 0.78 were observed in only the group treated with 50 mM salt solution. This result indicated that the plants in the other treatments were all subjected to stress since the F_v_/F_m_ values were less than 0.78 only when plants grow under stress^46^. In our experiment, the values of Fv/Fo and Fv/Fm under salt stress were lower than under alkali stress. We also found that salt and alkali stress both induced a decrease in PI values, indicating that plant vitality and PS II function were somewhat suppressed under severe stress^47^. Comparing the rate of change in Fv/Fm and PI in each treatment, we found that PI declined more than Fv/Fm which is in agreement with the report that PI is much more sensitive than Fv/Fm by PDR van Heerden^48^. It is generally believed that higher values of Vj indicate decreased plastoquinone levels^49^. In our results, Vj was highest in Goji berry treated with 300 mM salt and alkali solution. In addition, the value of Vj under alkali stress was higher than that under salt stress. The increase in Vj indicated that electron transfer activity to PSII is inhibited due to the decrease in PQ in the chloroplasts^49^. The high pH of the alkali stress might damage the photosynthetic machinery and primary electron acceptors, inhibiting the photochemical reaction and the activity of photosystem II^50^, which results in photoinhibition in the plant and the activation of photoprotection mechanisms that involve blocking electron transport. These changes cause the values of Fo and Fm to simultaneously increase and decrease, respectively, which may be inhibited by the electron transfer process. This can subsequently disrupt the photophosphorylation reaction resulting in the reduction of ATP synthesis and lead to a lower Vj under salt stress than under alkali stress. The augments in Sm, ABS/RC, ETo/RC and DIO/CSo under alkali stress were verified by the prompt reaction of photosynthetic machinery to stress^51^. The reasonable interpretation of this phenomenon may be the decrease in the efficiency of light energy conversion leading to a self-regulating photosynthetic mechanism that absorbs increased amounts of light energy and is used in the RC activities, thus reducing stress-induced damage.

The results of this experiment indicated that both stresses harmed the cell membrane, which was manifested by the increase in MDA, a product of lipid peroxidation. In addition, alkali stress induced a greater increase in the MDA content than salt stress (Fig. 5A), which indicated that alkali stress caused more damage to Goji berry than salt stress. The salt treatment also increased the CAT, SOD, and POD activity. SOD is the first line of defence against ROS in plants. SOD catalyses the reaction of 2O_2_^−^ + 2 H + → H_2_O_2_ + O_2_, after which H_2_O_2_ is broken down by POD and CAT^52^. Therefore, it is reasonable to assume that the increase in MDA content under both stresses results in ROS accumulation. Compared with the variations in the MDA content, the CAT, SOD, and POD activities under the salt treatment present a parallel trend with MDA, and the increase in CAT, SOD, and POD activities likely improves the resistance to salt stress by eliminating ROS, which can lead to lipid membrane peroxidation. We found that the critical alkalinity level for Goji berry is 200 mM because at this point the CAT, SOD, and POD activities started to decrease under alkali stress. The alkali-induced increase in CAT, SOD, and POD activities suggested a reduced ability to eliminate ROS. ROS might initiate membrane lipid peroxidation, thereby resulting in reduced membrane lipid unsaturation and membrane protein polymerization and leading to increased membrane permeability. Thus, the accumulation of ROS may have negative effects on cellular tissues and antioxidant system mechanisms, thereby leading to increased mortality in Goji berry. The above results indicate that alkali stress induces greater damage to the antioxidant system than salt stress.

According to the quantitative analysis of the tolerance of Goji berry under salt stress and alkali stress, we found that under the conditions of this experiment, the optimal salt concentration for Goji berry growth ranged from 0 to 246.3 ± 2.1 mM, whereas the optimum alkali concentration ranged from 0 to 126.7 ± 2.7 mM. The relationship between the soil salt content and soil water content was fitted based on the salinity (NaCl) and alkalinity (NaHCO_3_) thresholds of Goji berry, and the soil water content required to maintain normal growth was determined according to the regression curve. The minimum soil water content to cultivate Goji berry should be 16.22% and 23.37% under mild and moderate salt stress in soils, respectively, and 29.10% and 42.68% under mild and moderate alkali stress, respectively (Fig. 6). In areas with mild and moderate salinity, whether the Goji berry needs to be irrigated or not can be roughly determined according to this method. In addition, whether Goji berry is suitable for planting in the area can be estimated based on the actual conditions, such as soil salinity and long-term achievable soil moisture content. However, because of the limitations of this experiment, the drought stress caused by the soil water content and the complex species of salt or alkali were not considered, and the method is not suitable for the soil with extremely low or high contents of salt or alkali. An extremely low content of salt or alkali corresponds to an extremely low soil water content, which will cause drought stress on plants and inhibit the growth of plants. An extremely high salt or alkali content corresponds to an extremely high soil water content, which might damage plants; moreover, soil water content is difficult to maintain in actual production.

## Conclusion

In summary, salt and alkali stress had negative effects on the photosynthesis, Chl fluorescence, and physiology of Goji berry. Both salt and alkali stress reduced all the indicators, and alkali stress was more harmful to Goji berry than salt stress under the same solution concentration. Based on the regression analysis, the thresholds of salt tolerance and alkali tolerance of Goji berry were determined. These thresholds were then used to determine the optimal water content of Goji berry to maintain growth. This research provides insights into the damaging effects of salt stress and alkali stress in general and in Goji berry in particular, and the results of this study can be used as a basic guide for the production and irrigation requirements of Goji berry in Qinghai. Further studies are needed to understand the coupling of multiple salt complex stresses and drought stress as well as the comprehensive relationship between soil salt content and soil water content.

## Methods

### Plant material, growth conditions and stress conditions

Goji berry seedlings were collected from the Academy of Agriculture and Forestry Sciences, Qinghai University, China. The seedlings were used in a pot experiment that was conducted under rain-protected and ambient conditions in the greenhouse of Qinghai Nuomhon Farm in July 2016. Prior to the planting of seedlings, a pre-test was conducted to determine the optimum moisture content of Goji berry. Twelve well-grown plants were divided into four groups and the water contents of the four groups were set at the following four levels: 10.7%, 12.9%, 15.1%, and 17.2%. The soil water content of the pots was determined by the weighing method, in which the pots were sealed with plastic wrap, weighed and replenished with water at 8:00 every day. The optimal moisture content was determined by monitoring the photosynthetic indices of Goji berry in the four treatments after the pre-test. The optimal moisture content value in Nuomhon area was at the field capacity of 16.9%.

Three weeks after cultivation, uniform one-year-old seedlings (70 cm in height) were individually transplanted into polyethylene pots (36 cm × 34.5 cm × 40 cm), with each containing 10 kg of non-saline sandy soil (0~60 cm) collected from a Goji berry field. This soil was air-dried and passed through a 2-mm sieve to remove large stones, litter, and plant fragments. This soil has a gravimetric water content at the field capacity of 7.5%, a field capacity of 22.7%, a bulk density of 1.6 g cm^−3^, a pH of 7.1 and a total humus percentage of 2%. The ion concentrations were as follows (mg g^−1^): CO_3_^2−^, 0.02; HCO_3_^−^, 0.07; Cl^−^, 0.13; SO_4_^2−^, 0.36; Na^+^, 0.15; Ca^2+^, 0.03; K^+^, 0.05 and Mg^+^, 0.02.

The seedlings were divided into 9 groups, among which one group was used as a control, 4 groups were used for the salt stress experiments and 4 groups were used for alkali stress experiments. The plants for the different stress experiments were irrigated with half-strength Hoagland and Arnon’s solution supplemented with 0 (control), 50, 100, 200 or 300 mM NaCl (SS) and the same four NaHCO_3_ (AS) treatments. Each treatment included six replicates. The stress concentration was treated by adding 50 mM per day until the predetermined concentration was reached to prevent exciting the reaction. All pots were placed in a greenhouse with plastic trays underneath to prevent salt and alkali stress caused by water supplementation. To obtain the response of different stresses in the natural environment, the pots were moved outdoors every morning for natural light irradiation and were covered with a moveable shelter to avoid the impact of rainfall. Additionally, the average day and night temperatures of the greenhouse were 25.5 ± 1.5 °C and 18.5 ± 1.5 °C, respectively, and the photoperiod ranged from 10 to 12 h. The water deficit was calculated by weight, the soil was replenished with deionized water every day, and the moisture was maintained at 16.9% during the course of the experiment.

### Chlorophyll pigment content, photosynthetic performance and chlorophyll fluorescence

Chlorophyll a, chlorophyll b and carotenoidss were extracted from fresh fully expanded leaves (1.0 g) with a mixture of acetone, ethanol, and water in a volumetric ratio of 4.5:4.5:1. The absorbance values of the extract were measured using a spectrophotometer (UV-7504; Shanghai Precision Instrument Co., Ltd., Shanghai, China) at wavelengths of 440, 663 and 645 nm. The concentrations of Chl a, Chl b and Cars were determined by the following formulas^53^:

1 $${\rm{Chl}}\,{\rm{a}}=(12.21{{\rm{D}}}_{663}-2.96{{\rm{D}}}_{645})\times {\rm{V}}\div1000{\rm{W}}$$

2 $${\rm{Chl}}\,{\rm{b}}=(22.88{{\rm{D}}}_{645}-4.67{{\rm{D}}}_{663})\times {\rm{V}}\div1000{\rm{W}}$$

3 $${\rm{Cars}}=[4.70{{\rm{D}}}_{440}-0.27(8.05{{\rm{D}}}_{663}+20.29{{\rm{D}}}_{645})]\times {\rm{V}}\div1000{\rm{W}}$$

D_440_, D_663_ and D_645_ are absorbance at 440, 663 and 645 nm, respectively. V is the extraction volume (ml), W is the leaf weight (g) and Chl (a + b) is the sum of the Chl a content and Chl b content.

The net photosynthetic rate (P_N_), stomatal conductance (g_s_), transpiration rate (E) and intercellular CO_2_ concentration (C_i_) of leaves were estimated at 8:00, 10:00, 12:00, 14:00, 16:00 and 18:00 on mature leaves using a LI-6400 portable open-flow gas exchange system (LI-COR Biosciences, Lincoln, NE, USA) at 20 days after stress treatment. The values obtained for P_N_, g_S_, E and C_i_ were expressed as μmol m^−2^ s^−1^, mol m^−2^ s^−1^, mol m^−2^ s^−1^and μmol mol^−1^, respectively. The photosynthetically active radiation was 1 mmol m^−2^ s^−1^ (saturation light). The ambient CO_2_ concentration was 335 ± 15 μmol mol^−1^, the air temperature was 20 ± 1.5 °C, and the air humidity was approximately 40 ± 5%. Measurements were repeated five times for each of five blades per pot, and the average values were recorded. The water use efficiency (WUE) was calculated according to the following formula:

4 $${\rm{WUE}}=\frac{{P}_{N}}{E}$$

where WUE is the water use efficiency and P_N_ (μmol m^−2^ s^−1^) and E (mmol m^−2^ s^−1^) are the net photosynthetic rate and transpiration rate, respectively.

Chl fluorescence was measured using a LI-6400 system with a 6400-40 leaf chamber fluorometer (LI, Washington, DC, USA). Seedlings were preserved in the dark for approximately 30 min, before recording the fluorescence of the blades, which were the same blades for which the photosynthetic parameters were determined. The basal nonvariable Chl fluorescence level (F_o_) was determined by a modulated light, which was maintained at a sufficiently low intensity (<1 μmol m^−2^ s^−1^) so that notable variations in fluorescence did not occur. The maximum fluorescence induction (F_m_) was estimated by a 0.8-s saturation pulse at 4200 μmol m^−2^ s^−1^ on dark-adapted leaves. The variable fluorescence (F_v_ = F_m_ − F_o_) was then calculated. Measurements of the maximum quantum yield of PSII (F_v_/F_m_) were obtained by application of a saturation light pulse. A number of variables were measured, including the absorption energy flux (ABS), the performance index of intersystem electron acceptors (PI), the electron transport (ETo), the relative variable fluorescence at the J step (V_j_), the trapping (TRo), and other Chl fluorescence parameters.

### Antioxidant enzyme assays

The MDA content was measured by following the method described by Xu *et al*.^54^ SOD activity was assayed by monitoring the capability of the enzyme to inhibit the photochemical reduction of nitro blue tetrazolium (NBT)^55^. POD activity was analysed by measuring the increase in absorbance at 470 nm recorded 40 s after adding H_2_O_2_^56^. CAT activity was determined based on the amount of H_2_O_2_ consumed during the reaction^57^.

### Statistical analysis

All data were subjected to a one-way analysis of variance (ANOVA), and the mean differences were compared by the least significant difference (LSD) test at the P ≤ 0.05 level with Duncan’s multiple range test (DMRT). Pearson’s correlation coefficients were also analysed between the parameters. Data are expressed as the mean ± standard error (SE) in the figures and tables. Statistical analyses were performed with SPSS v. 19.0 software (SPSS Inc., Chicago, IL, USA). All data were plotted using OriginPro 2018 (Originlab Corporation Northampton, MA, USA).
